# Generating high repetition rate X-ray attosecond pulses in a diffraction limited storage ring

**DOI:** 10.1038/s41598-023-41118-0

**Published:** 2023-08-28

**Authors:** Weihang Liu, Yu Zhao, Yi Jiao, Sheng Wang

**Affiliations:** 1 Institute of High Energy Physics, Chinese Academy of Sciences, Beijing, 100049 China; 2 Spallation Neutron Source Science Center, Dongguan, 523803 China

**Keywords:** Techniques and instrumentation, High-harmonic generation

## Abstract

To steer and track electron motion in atoms, molecules, and nanostructures, light pulses with attosecond duration and high repetition rate are required. In this paper, we use the angular dispersion-induced microbunching scheme and a few-cycle laser within a straight section (a few meters) of a diffraction-limited storage ring to generate a coherent high-flux attosecond pulse in the water window region. Simulation results based on the Southern Advanced Photon Source indicate that the proposed method can generate a chirp-free Fourier transform limited pulse with a minimum duration of 50 as, a maximum repetition rate of a few MHz, and a maximum average flux of about $4.4\times 10^{11}$ photons/s/1%Bw.

## Introduction

Attosecond, which is the natural time scale of electron motion in atomic and molecular systems, has significant implications for several cutting-edge fields, such as quantum physics, biology, chemistry, and medicine. To advance scientific research in these fields, the development of high-flux, high repetition rate attosecond light sources is considered critical^1^.

So far, only two types of light sources, namely high-harmonic generation (HHG) based light sources and free electron lasers (FELs), have been employed to generate attosecond pulses in experimental studies. Currently, the HHG sources hold the record for the shortest attosecond soft X-ray pulse, at approximately 43 attoseconds^2^. Furthermore, FEL sources have successfully produced attosecond soft X-ray pulses with duration of around 280 attoseconds^3^. It should be noted, however, that although HHG sources can produce X-rays, their conversion efficiency in this wavelength range is considerably low. This results in a reduced photon flux (about $10^3$ photons/pulse/1%Bw for soft X-ray^4,5^), which does not meet the requirements of most experiments^6^. On the other hand, attosecond experiments based on FELs need to adopt the electron beam shaping method^3^, which cannot be easily shared among users with different research needs, thus making the experiment time prohibitively expensive.

Unlike the above two types of light sources, the storage ring-based light source is a stable, high repetition rate, a multi-user light source that has been at the forefront of high-brilliance experiments. It has evolved from the third generation to the fourth generation, also known as the diffraction-limited storage ring (DLSR)^7–9^, increasing brilliance by more than two orders of magnitude. Due to these advantages, the ability to achieve attosecond pulses in DLSRs would make them very attractive. By achieving high-brilliance and providing high-flux and high repetition rate attosecond pulses, DLSRs can make great contributions to the development of attosecond science.

In DLSR, the natural light pulse is typically in the range of 10 ps to 100 ps due to the stretching of the electron beam length to reduce the collective effects. This pulse duration is more than five orders of magnitude different from the attosecond, and obtaining an attosecond pulse by shortening the electron beam length or by slicing only a fraction of the electrons for radiation means a huge reduction in flux, making it very difficult to achieve a high-flux attosecond pulse in a DLSR.

Recently, Hwang, et al.^10^ adopted the echo-enabled harmonic generation (EEHG) scheme^11–17^ to generate isolated attosecond pulses in the BESSY II storage ring by using two adjacent straight sections to arrange the EEHG structure. Local microbunching of sub-femtosecond duration is induced in the electron beam by modulation with a few-cycle laser. The simulation shows that soft X-ray pulses of 290 as FWHM duration with a repetition rate of up to 6 kHz and a single pulse flux of $10^6$ photons/pulse/1%Bw can be achieved.

However, to produce such an attosecond pulse, the energy modulation intensity is about 8 times the initial energy spread of the beam. This results in a large increase in the beam energy spread, by a factor of about 6. In addition, the EEHG requires two laser modulations, and the second laser is a short-duration laser with few cycles, which makes it very difficult to synchronize the two lasers with the electron beam. Furthermore, the EEHG layout occupies two straight sections but supports only one experimental station.

In this paper, we propose a novel method that combines angular dispersion-induced microbunching (ADM)^18–21^ and a few-cycle laser to generate attosecond pulses in a DLSR. The ADM method takes advantage of the low emittance in the vertical direction of the storage ring, which can achieve microbunching in a single modulation and can be implemented in a straight section. In addition, the ADM method requires less laser power to achieve the same performance as EEHG and other similar methods^22–28^.

Note that after a modulation, the next modulation cannot be performed immediately due to the reduced beam quality. Therefore, the repetition rate of the proposed method (and other similar methods) is limited by the recovery time of the beam quality compared to the steady-state microbunching (SSMB)^29,30^ or the reversible modulation method^20^. However, there is no suitable method to implement the SSMB and the reversible modulation method in a normal DLSR. To further increase the repetition rate, considering that the few-cycle laser modulates only a small fraction of the electron beam, we appropriately design the time delay between the laser and beam to modulate the fresh part of the electron beam at different turns. In this way, the repetition rate can be increased to the MHz range.

In addition, the combination of ADM and a few-cycle laser has the following additional advantages: Firstly, the radiation pulse generated by the microbunched beam is longitudinally coherent and has no chirp, making it close to the Fourier transform limit (FTL). This characteristic distinguishes it from the pulse generated by an HHG source, which has a natural chirp and requires additional chirp compensation elements (such as inert gases or metals) to approach an FTL pulse^31^. Secondly, the  100 ps electron beam length of the storage ring provides a large arrival time tolerance for the few-cycle laser. Thirdly, since the modulation interval of the few-cycle laser is only about one ten-thousandth of the beam length, the perturbation of the beam parameters by a single modulation is minimal. As a result, the radiation brilliance of other insertion devices (IDs) is not affected for the low repetition rate case.

To demonstrate the performance, we apply the proposed method to the Southern Advanced Photon Source (SAPS)^32^, as an example, which is a DLSR in the design phase and planned to be built in the Guangdong Province of China, adjacent to the China Spallation Neutron Source^33^.

**Figure 1:**
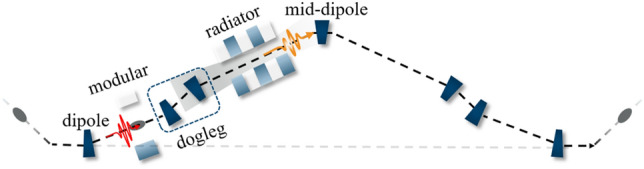
Layout of the ADM section in one of the straight sections of SAPS.

The ADM structure, located in one of the straight sections of SAPS as illustrated in Fig. 1, initially couples the electron beam in both transverse and longitudinal directions using a vertical dipole. Subsequently, the beam undergoes energy modulation through interaction with a short wiggler (called a modular) and a few-cycle laser. Finally, a dogleg consisting of two vertical dipoles with equal strength but opposite deflection angles imparts transverse and longitudinal dispersion that converts the energy modulation to density modulation, resulting in microbunching and a large local peak current in the electron beam. The modulated beam then passed through an undulator (called a radiator) to generate a coherent attosecond pulse. After this process, the radiated electron beam passes through four dipole magnets that are specially designed in position and strength to eliminate the vertical dispersion and allow the electron beam to return to the ring.

## Optimization and performance

In the ADM method, the conditions for generating microbunching are^18^,

1 $$\begin{aligned} \left\{ {\begin{array}{l} {1+h r_{56} = 0} \\ {r_{56} +b\eta =0} \\ \end{array} } \right. , \end{aligned}$$

where *b* is the deflection angle of the first dipole, $\eta$ and $r_{56}$ are the vertical and longitudinal dispersion of the dogleg, respectively. The parameter *h*, also known as the local energy chirp, is determined by the energy modulation intensity *A* and the wave number $k_L$ of the seed laser:

2 $$\begin{aligned} h =A k_L. \end{aligned}$$

For a given set of laser parameters and operating under the ADM optimal conditions (Eq. 1), the dogleg vertical dispersion $\eta$ is constrained by the geometric layout of the ADM structure. As shown in Fig. 1, all dipole elements (each with a length of $L_b$, except for the mid-dipole which has a length of $2L_b$) are symmetrically distributed in a straight section (with a length of *L*) of the ring, ensuring that the beam orbit returns to the horizontal plane.

To find the boundaries of the $\eta$, assuming a truncation of the dipole deflection angle $\theta$ up to the second order term, the $r_{56}$ and $\eta$ ($>0$) of the dogleg can be expressed as follows:

3 $$\begin{aligned} \left\{ \begin{array}{l} {(L_2+L_b)\theta = \eta } \\ {\frac{1}{3}(3L_2+2L_b)\theta ^2 =r_{56}} \end{array}\right. , \end{aligned}$$

here, $L_2$ represents the distance between the two dipoles of the dogleg.

Given the values of $r_{56}$ and $\eta$, Eq. (3) can be solved to determine two sets of $\theta$ and $L_2$. The solution with the smaller absolute value of $\theta$ is selected:

4 $$\begin{aligned} \left\{ \begin{array}{l} {\theta = \frac{-3\eta +(-12 L_b r_{56}+9\eta ^2)^{1/2}}{2L_b}} \\ {L_2 = \frac{-6L_b r_{56}+3\eta ^2+(-12 L_b r_{56}+9\eta ^2)^{1/2}}{6r_{56}}} \end{array}\right. . \end{aligned}$$

To guarantee that the total space occupied by the dipole, modulator, dogleg, radiator, and the half mid-dipole is less than *L*/2, the inequality $0 \le L_2 < (L/2 - L_3 - 4L_b - L_1)$ must hold, where $L_1$ and $L_3$ are the distances between the first dipole of the ADM section and the first dipole of the dogleg, and between the second dipole of the dogleg and the middle dipole of the ADM section, respectively. By substituting the expression for $L_2$ from Eq. (4) and ensuring a real result for the square root, it can be deduced that $\eta$ must satisfy the following condition:

5 $$\begin{aligned} (3L_b/2|h|)^{1/2}\le \eta < (3L_{eff}/2|h|)^{1/2}, \end{aligned}$$

where $L_{eff} = (L-2L_1-2L_3-5L_b)^2/(3L-6L_1-6L_3-17L_b)$. Note that the upper bound of $\eta$ increases as the length of the straight section grows, while the finite length of the straight sections in the storage ring directly limits the optimization space of $\eta$.

After the modulation with a few-cycle laser, a current spike is generated in the electron beam. When Eq. (1) is satisfied, the local peak current *I* can be expressed as^34^,

6 $$\begin{aligned} I(s)/I_0=1+2\sum _{m=1}^{\infty }f(m)\cos (k_Lms), \end{aligned}$$

with

7 $$\begin{aligned} f(m)=\frac{0.67}{m^{1/3}}\exp [-\frac{1}{2}(mk_L\eta )^2\gamma _y\epsilon _y], \end{aligned}$$

where $I_0$ is the peak current of the beam before modulation, *m* is the harmonic number of the seed laser, $\gamma _y$ and $\epsilon _y$ are the vertical Twiss function and emittance, respectively. For large $I/I_0$, one needs a large *f*(*m*), which implies small $\eta$ and $\epsilon _y$.

To characterize the intensity of the microbunching, we adopt the local bunching factor for the harmonic *n*, which is defined as^35^,

8 $$\begin{aligned} b_n(s)=\frac{\int _{s-\pi /k_Ln}^{s+\pi /k_Ln}I(\tau )/I_0\exp (-ik_Ln\tau )d\tau }{\int _{s-\pi /k_Ln}^{s+\pi /k_Ln}I(\tau )/I_0d\tau }. \end{aligned}$$

With Eqs. (6) and (7), we obtain the explicit expression for the local bunching factor of the ADM:

9 $$\begin{aligned} b_n(s)=\frac{2\sum _{m=1}^{\infty }f(m)\sin (m\pi /n)\frac{m\cos (k_Lms)+in\sin (k_Lms)}{n^2-m^2}}{\pi /n+2\sum _{m=1}^{\infty }f(m)\sin (m\pi /n)\frac{\cos (k_Lms)}{m}}\exp (-ik_Lns). \end{aligned}$$

**Figure 2:**
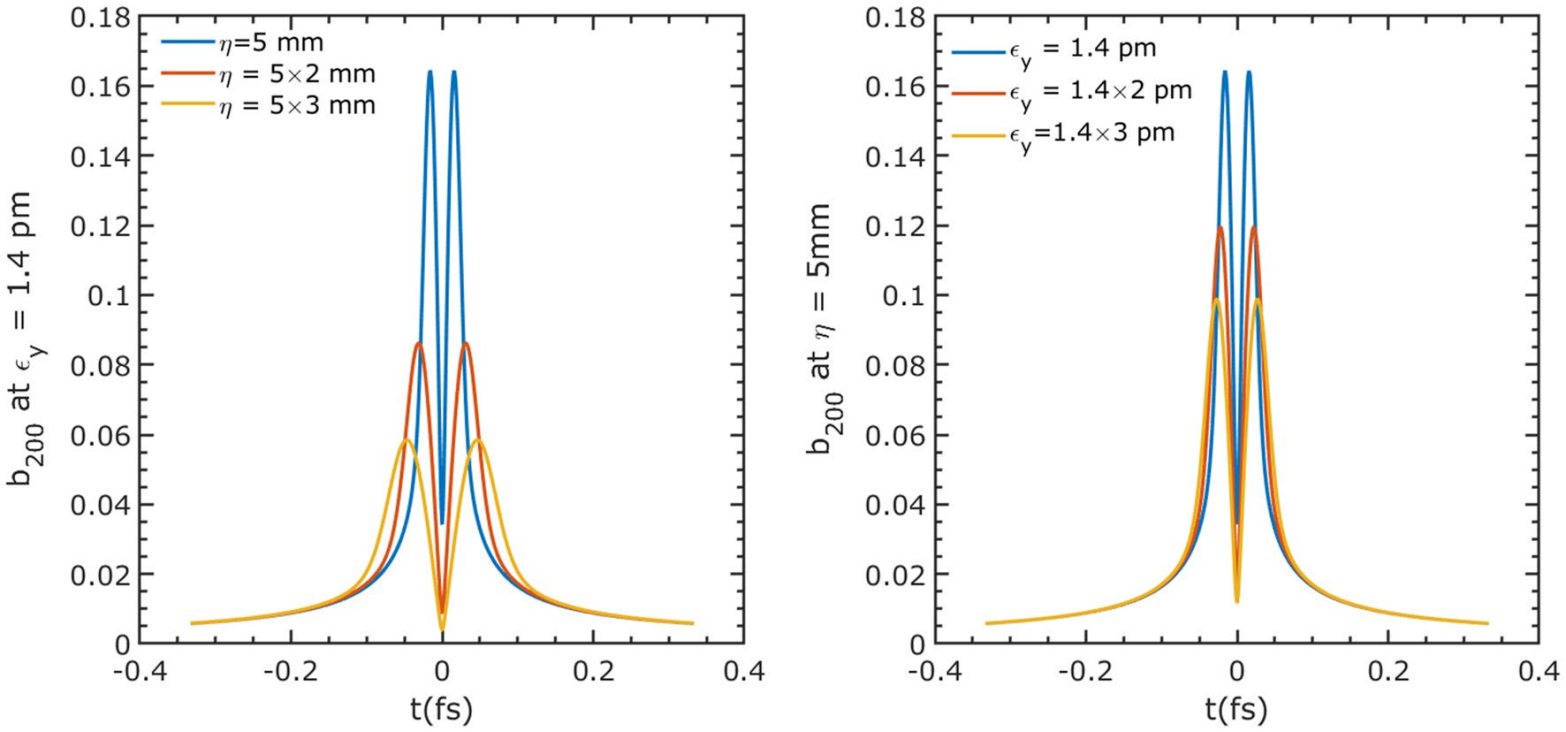
Local bunching factor of harmonic 200 for different $\eta$ and $\epsilon _y$: low $\eta$ and $\epsilon _y$ resulting large bunching factor and the bunching factor is more sensitive to $\eta$ compared to $\epsilon _y$.

For a given laser wavelength of 800 nm, we calculate the local bunching factor at a harmonic number of 200 (corresponding to the wavelength at 4 nm) under different dogleg dispersion and vertical emittance. The results are shown in Fig. 2. The bunching factor has two symmetrical peaks, and the peaks increase with decreasing vertical emittance and dogleg dispersion. The change rate of the bunching factor is more sensitive to dispersion compared to emittance. Moreover, the bunching factor with sufficient values covers an interval of about 0.4 fs, which guarantees the generation of an attosecond pulse.

In order to obtain high radiation power, a sufficiently large $I/I_0$ and local bunching factor are required. They all need low $\eta$ and $\epsilon _y$. Note that for a given energy modulation intensity *A*, the acceptable range of $\eta$ is determined by Eq. (5). All parameters of the dipole in the ADM section are determined by Eqs. (1)–(4) for a given $\eta$ in the acceptable range. When the betatron coupling of the ring is defined, all dipoles of the ADM section specify the vertical emittance $\epsilon _y$. Thus $\epsilon _y$ can be considered as a function of $\eta$.

To obtain a small $\epsilon _y$, it is necessary to control the betatron coupling to a small level, which we assume to be 0.4%. This can be achieved using a suitable beam control technique^36^. Furthermore, to take into account the intrabeam scattering (IBS) effect, we adopt the completely integrated modified Piwinski approximation^37^ method, which incorporates the vertical dispersion and betatron coupling to calculate the equilibrium parameters of the ring.

In SAPS, the straight section length $L =6$ m, and to provide enough space for the modular and the radiator, we set $L_1$ and $L_3$ to 0.7 m and 1 m, respectively. The dipole length $L_b$ is 0.2 m and the laser wavelength is 800 nm. Other SAPS parameters are listed in Table 1.

**Table 1:** Main parameters of the SAPS.

Parameter	Value	Unit
Energy	3.5	GeV
Circumference (C)	810	m
Natural emittance	33.4	pm
Energy spread (w/o IBS)	0.11	%
Bunch number	405	
Bunch charge	3.33	nC
Bunch length	30	mm
Length of straight section	6	m
Damping time $\tau _x/\tau _y/\tau _z$	14/21/13.8	ms
$\gamma _y$	0.22	1/m
Peak current $I_0$	13.3	A
Momentum compaction factor $\alpha _P$	$2.5\times 10^{-5}$	
Chromaticities $\xi _x/\xi _y$	5/5	

**Figure 3:**
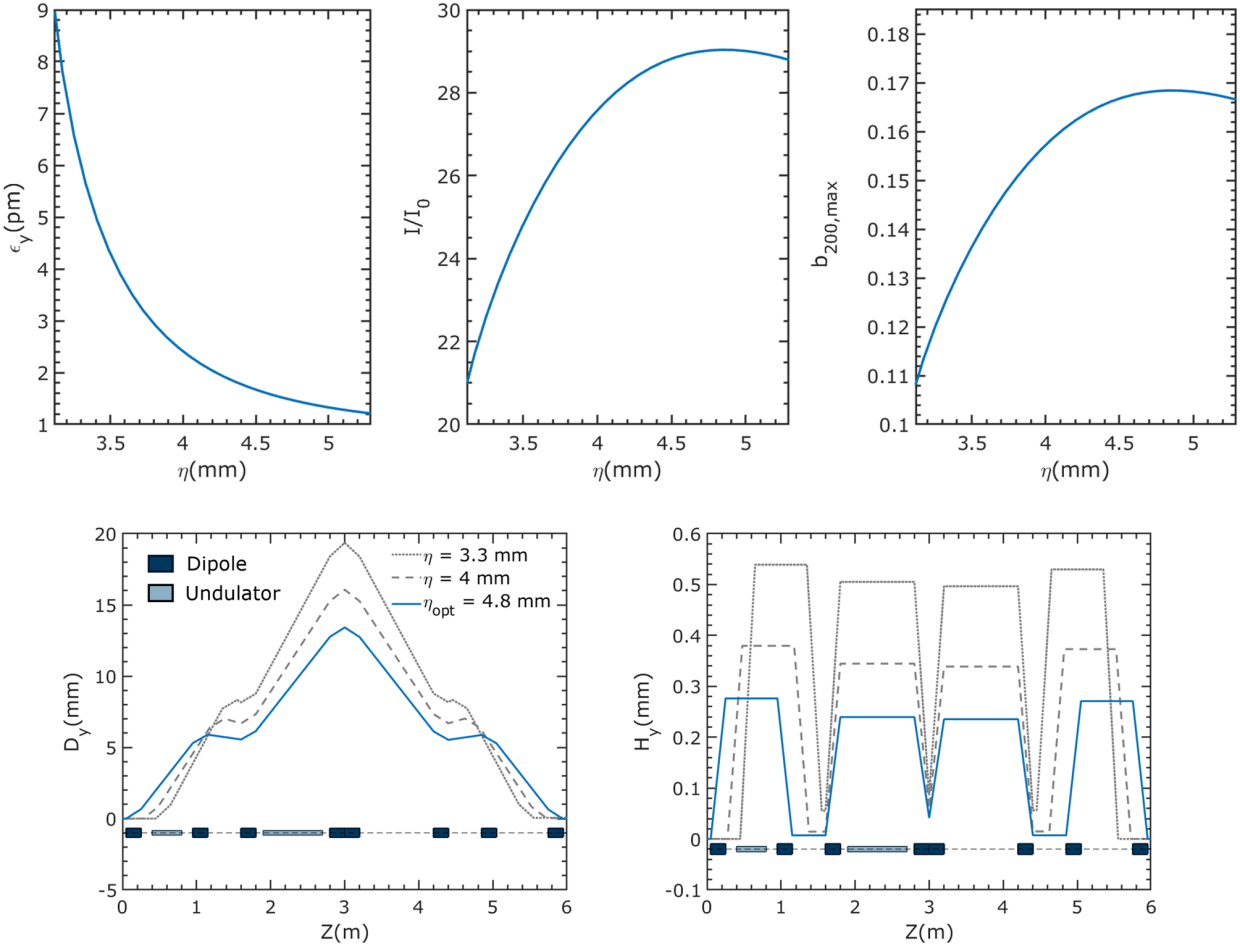
Top: Vertical emittance, peak current and peak local bunching factor for different $\eta$. Bottom: Vertical dispersion ($D_y$) and vertical chromatic *H* function ($H_y$) of the ADM section under different $\eta$. The element distribution shown in the figure is only for the case of $\eta$ = 4.8 mm. In other cases, the position of some elements is slightly different. The undulator parameters are given in Table 2.

Taking the energy modulation intensity *A* as 0.4%, which is about 3 times of the initial beam energy spread with IBS, this value defines the acceptable $\eta$ range as (3.1 mm, 5.3 mm). In this range, the vertical chromatic *H* function decreases with increasing $\eta$, leading to a decrease in the vertical emittance with increasing $\eta$. The corresponding $I/I_0$ and the peak of the local bunching factor have optimum values obtained at $\eta = 4.8$ mm of 29 and 0.17, respectively, as shown in Fig. 3. Additionally Fig. 3 presents the vertical dispersion function ($D_y$) and the vertical chromatic *H* function ($H_y$) of the ADM section under different $\eta$. It is evident from the figure that $\eta$ exhibits a negative correlation with both vertical dispersion and $H_y$. At the optimum, the equilibrium emittances and the energy spread of the ring are $\epsilon _x = 100$ pm, $\epsilon _y = 1.4$ pm and 0.134%, respectively. The optimal parameters of the ADM section are given in Table 2.

**Table 2:** The optimal parameters of the ADM elements.

Dipole	Value	Unit
Bending angle	6.6	mrad
Dogleg		
Bending angle	7.4	mrad
Distance between dipoles	0.45	m
Modulator		
Peak field	0.52	T
Period length	0.4	m
Radiator		
Peak field	0.996	T
Period length	0.04	m
Seed laser		
Pulse energy	0.8	mJ
FWHM pulse duration	3.4	fs

### Performance

**Figure 4:**
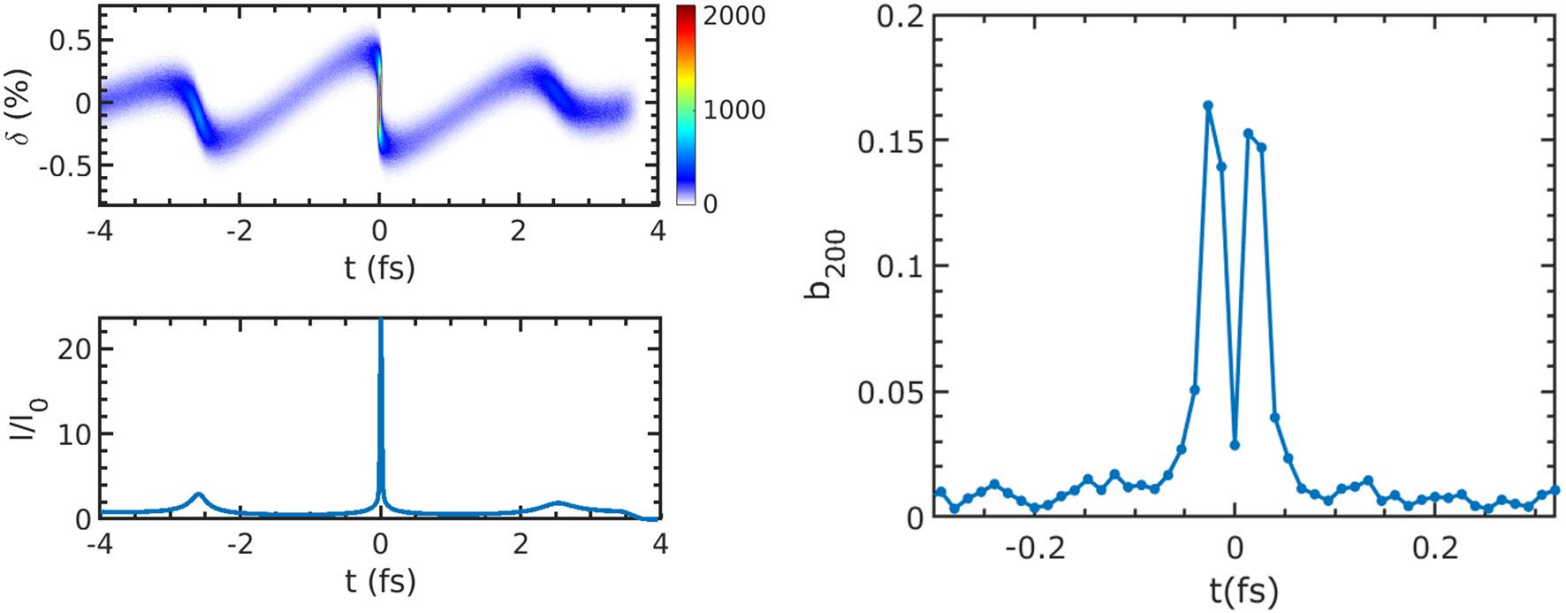
longitudinal phase space, $I/I_0$ and local bunching factor of the beam after density modulation.

We adopted GENESIS^38^ and ELEGANT^39^ codes to perform the laser modulation and beam tracking in the ADM beamline. After interacting with a near single cycle 800 nm laser, which is available with the state of the art technology^40^, within a 0.4 m long wiggler with one period, the electron beam passes through the dogleg, then a microbunching is generated. The local current is amplified by 24 times and the peak local bunching factor is about 0.16, as shown in Fig. 4, which are in a great agreement with the theoretical optimal results.

**Figure 5:**
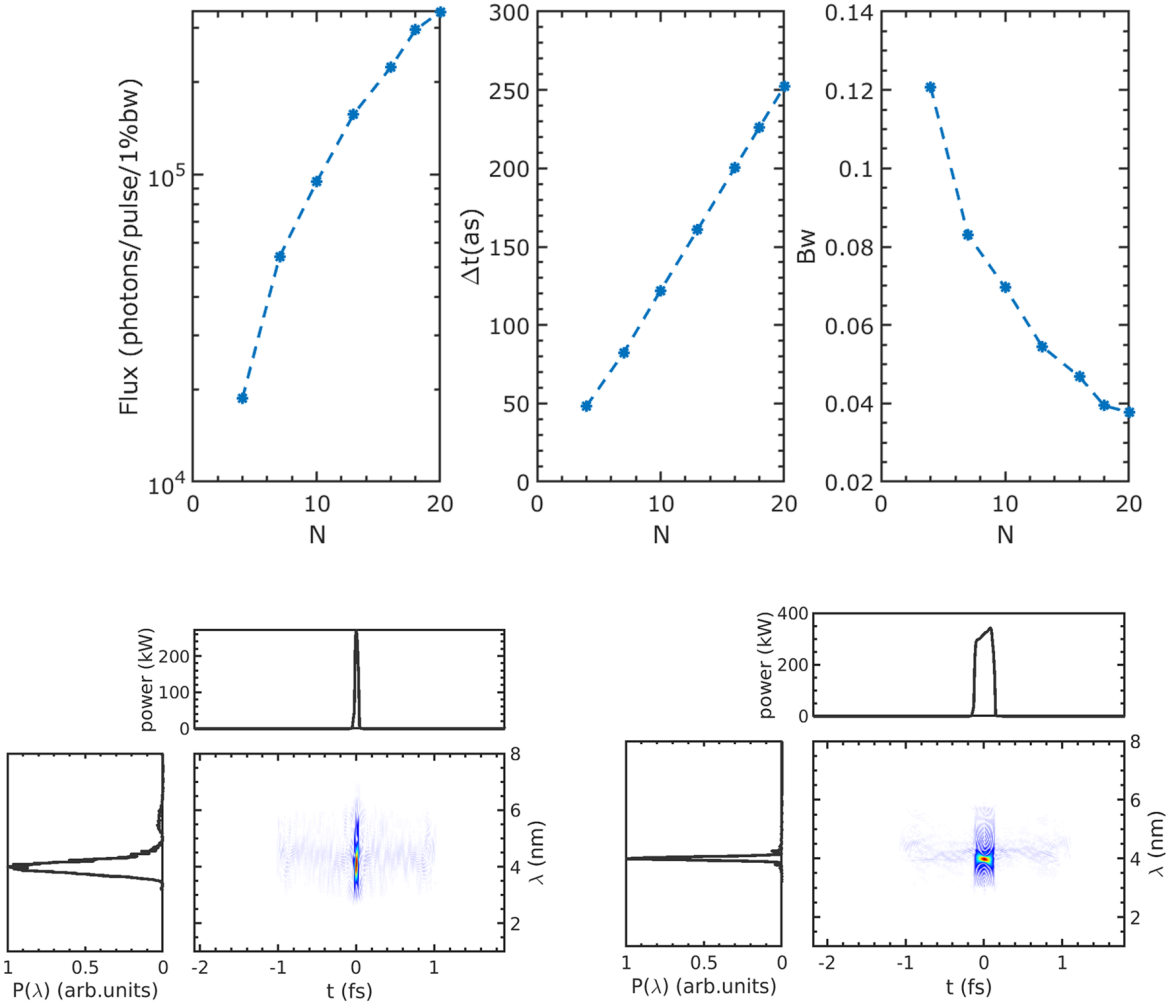
Top: Performance of the radiation generated by the radiator with varying period numbers. From left to right: Flux per pulse, FWHM pulse duration, and FWHM bandwidth. Bottom: Wigner distribution and time spectrum profile for the output of $N = 4$ (left) and 20 (right), respectively.

The modulated beam is capable of producing a coherent pulse with a wavelength of 4 nm and varying FWHM pulse duration $\Delta t$ within the radiator, depending on the period number *N*. The radiation simulations are carried out by GENESIS with a self-consistent method^41^. Figure 5 shows that $\Delta t$ is directly proportional to *N*. In order to achieve a pulse duration of less than 100 as, it is necessary to ensure that *N* is less than 10. However, small *N* results in lower photon flux and larger FWHM bandwidth (Bw). For instance, when $N = 4$, the pulse duration can be as short as 50 as, but the flux is more than an order of magnitude lower than that of $N = 20$. The FWHM pulse duration, Bw and photon flux of three cases with different *N* are shown in Table 3 for comparison.

**Table 3:** Performance of the radiation for three different periods.

Period number	$\Delta t$ (as)	Bw	Flux ($10^5$photons/pulse/1%Bw)	$\Delta t \Delta E$ (fs eV)
4	50	0.121	0.19	1.88
10	122	0.07	0.95	2.65
20	252	0.038	3.39	2.97

It is well known that attosecond pulse form HHG has an intrinsic chirp called atto-chirp^42^. This chirp limits the duration of the pulse and needs additional compensation methods. Typically, the atto-chirp is compensated by shooting the attosecond pulse through thin metal foils or neutral gases depending on the wavelength of the pulse^31^. The transmittance of these materials is not perfect, pulse energy will be lost in the material which may induce thermal loading problems and limit the repetition rate of the attosecond pulse. In contrast, the proposed method generates chirp-free attosecond pulses, as evidenced by the Wigner distributions of the pulses shown in Fig. 5. In other words, these pulses are Fourier transform limited. As shown in Table 3, the time-bandwidth products are close to the Fourier transform limited ($\thicksim$1.83 fs$\cdot$eV).

**Figure 6:**
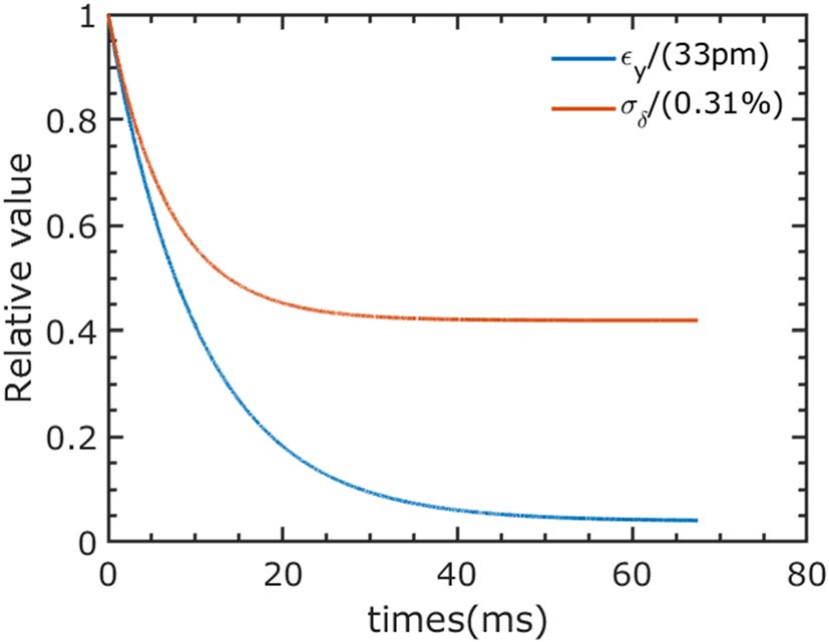
The vertical emittance and energy spread evolution of the modulated local beam after the radiation.

Note that the few-cycle laser modulates only a small portion of the beam, about 0.01%. The vertical emittance and energy spread of this modulated portion increase from 1.4 pm to 33 pm and from 0.134% to 0.31%, respectively. As shown in Fig. 6, these parameters quickly return to their equilibrium values with the help of radiation damping. This happens within about four times the damping time, i.e., 60 ms.

Given the presence of 405 bunches in the ring, the repetition rate can reach up to 6.75 kHz, provided that each bunch is modulated only once in each 60 ms recovery period. The average flux for pulses of 50 as, 122 as, and 252 as FWHM duration are $1.28\times 10^8$, $6.4\times 10^8$, and $2.29\times 10^9$ photons/s/1%Bw, respectively.

## Improvement of repetition rate

When each bunch in the ring undergoes only one modulation during the 60 ms recovery period, the corresponding repetition frequency is 6.75 kHz. It is worth noting that during each modulation, only a small part of the beam (with beam length $\sigma _b$) is modulated by the few-cycle laser (with duration $\sigma _l$). If multiple modulations of different parts of the bunch are carried out during each recovery period, the repetition rate can be increased to a maximum of approximately $\sigma _b/\sigma _l$ times.

However, after each modulation, the particles undergo a transverse betatron oscillation and are affected by the momentum compaction effect so that the modulated particles have a path length variation after one revolution. The equivalent path length difference of the modulated electron beam can be estimated as^43^

10 $$\begin{aligned} \sigma _L=\sqrt{(\alpha _pC\sigma _{\delta })^2+(\pi \xi _x\epsilon _x+\pi \xi _y\epsilon _y)^2} \end{aligned}$$

where $\alpha _p$ is the momentum compaction factor, *C* is the circumference of the ring, $\xi _{x,y}$ are the chromaticities of the ring, $\sigma _{\delta }$ and $\epsilon _{x,y}$ are energy spread and emittances of the modulated beam. The estimated value of $\sigma _L$ is about 63 $\mu$m. Therefore, the required delay distance for the laser must exceed 63 $\mu$m.

**Figure 7:**
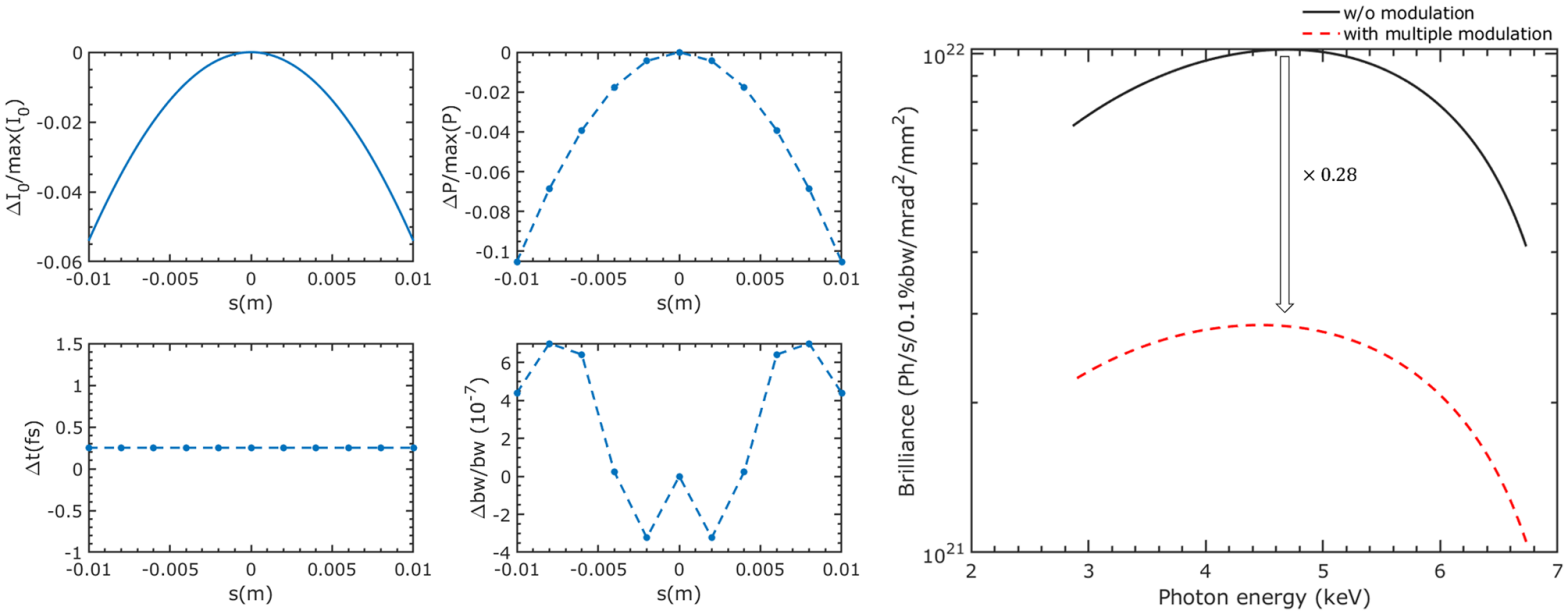
Left: Variation of peak current, peak radiation power, FWHM pulse duration, and FWHM bandwidth at different longitudinal positions in the beam. Right: Comparisons of brilliance between SAPS with and without ADM section, calculated under a 4.8 m length, *k* = 1.1, and 16 mm period undulator.

Furthermore, it is important to consider the Gaussian longitudinal distribution of the beam, as a delay distance that is too large can result in a decrease in local current. Figure 7 shows that when a delay length of 0.1 mm is selected and 200 modulations are performed on the beam, the corresponding reduction in local current is maximally 6%, and there is a decrease in radiation power of approximately 10%. This suggests that multiple modulations generate radiation pulses with a variation magnitude of less than 10%. In this way, the repetition rate can be increased to 1.35 MHz and the flux per second of the radiation pulses with pulse FWHM durations of 50 as, 122 as, and 252 as can reach $2.47\times 10^{10}$, $1.23\times 10^{11}$, and $4.4\times 10^{11}$ photons/s/1%Bw, respectively.

In addition, the degradation of the beam parameters of the modulated beam after multiple modulations is more severe than that after a single modulation. This degradation significantly affects the brilliance of other IDs. It is noteworthy that the beam parameters of the modulated part of the beam gradually recover to equilibrium within 60 ms following modulation. As a result, estimating the overall degradation of beam parameters can be challenging.

To simplify our analysis, we assume that the beam parameters of the modulated portion after a single modulation represent the parameters of the entire beam when calculating the brilliance change. As shown in Fig. 7, the highest brilliance of an ID in the ring decreased by approximately 72%. Although the actual decrease, which is less than 72%, is still significant and cannot be ignored. Therefore, with the proposed method, high repetition rate attosecond experiments cannot be conducted simultaneously with other high-brightness experiments.

To achieve a repetition rate of MHz, it is necessary to employ a few-cycle laser at the MHz level. The development of advanced laser technology has made it possible to achieve an ultra-short high-energy pulse laser with a repetition rate up to and even beyond 1 MHz using the optical parametric chirped-pulse amplification (OPCPA) scheme^44,45^.

## Discussion and conclusion

We propose a method that combines ADM and a few-cycle laser to generate a coherent attosecond pulse in a DSLR. Compared with the EEHG-based method, the proposed method can be applied to a single straight section with only one energy modulation, which makes it easier to synchronize the laser and electron beam and requires less laser power. The proposed method can achieve a repetition rate of 6.75 kH for the attosecond pulse during single modulation of a given electron beam. By introducing a suitable time delay between the laser and the beam, the modulation can be performed repeatedly on an electron beam, thereby increasing the repetition rate to 1.35 MHz.

Of course, the vertical dispersion introduced by the ADM section in the straight section breaks the periodicity of the storage ring and has some effects on the beam dynamics. The calculations show that both the dynamic aperture (DA) and the local momentum apertures (MA) are reduced. But, the DA reductions of less than 2% and the MA reductions of less than 25% have a negligible impact on the daily operation of the storage ring.

In addition, our work shows a linear relationship between the number of undulator periods and the attosecond pulse duration. Different pulse durations can be obtained by using segmented undulators and adjusting the gap to control the number of periods. However, the phase shift between different segments may cause a reduction in the radiated power, which deserves further investigation. Note that when calculating the pulse duration for the undulator with different periods, we did not consider the case of the undulator with less than 4 periods, because their fluxes are too low with the current ADM parameters. With higher laser power, increasing the energy modulation depth to $\thicksim$0.8% (6 times the initial energy spread) and using an undulator with only 2 periods, this method is able to produce a shorter pulse of $\thicksim$ 25 as (FWHM) with a flux of $\thicksim 10^4$ photons/pulse/1%Bw.

At a repetition rate of 1.35 MHz, the multi-modulation leads to significant degradation of the beam quality, resulting in a noticeable reduction of the ID brilliance. How to further compensate for this degradation is currently an open question. Nonetheless, one can mitigate the degradation of the electron beam by reducing the energy modulation intensity, but this also implies a reduction of the photon flux of the attosecond pulses. Further research is needed to find a more feasible solution.

## Data Availability

The data supporting the findings of this study are available from the corresponding author upon reasonable request.
